# Twelve-month outcomes in overweight/obese users with mental disorders following a multi-element treatment including diet, physical activity, and positive thinking: The real-world “An Apple a Day” controlled trial

**DOI:** 10.3389/fpsyt.2022.903759

**Published:** 2022-08-23

**Authors:** Laura Giusti, Valeria Bianchini, Annalisa Aggio, Silvia Mammarella, Anna Salza, Stefano Necozione, Alessia Alunno, Claudio Ferri, Massimo Casacchia, Rita Roncone

**Affiliations:** ^1^Department of Life, Health, and Environmental Sciences, University of L'Aquila, L'Aquila, Italy; ^2^Division of Internal Medicine and Nephrology, School of Internal Medicine—San Salvatore Hospital, Department of Life, Health, and Environmental Sciences, University of L'Aquila, L'Aquila, Italy; ^3^University Unit Rehabilitation Treatment, Early Interventions in Mental Health—San Salvatore Hospital, Department of Life, Health, and Environmental Sciences, University of L'Aquila, L'Aquila, Italy

**Keywords:** diet protocol, physical activity, metacognitive group intervention, cardiovascular risk, mental disorders, obesity, metabolic syndrome, psychopharmacological treatment

## Abstract

The present study aimed to evaluate the 12-month effectiveness of a real-world weight loss transdiagnostic intervention in overweight/obese participants affected by mental disorders under psychopharmacological treatment. We conducted a real-world, controlled, pragmatic outpatient trial. We allocated 58 overweight/obese adults under psychopharmacological treatment from a mental health outpatient unit and 48 overweight/obese adults from a cardiovascular prevention outpatient unit, and assigned them to an intervention or treatment usual as condition (TAU) enriched by life-style advice. Participants in both intervention groups took part in a diet programme (the modified OMNIHeart dietary protocol) and monitoring of regular aerobic activity. A brief group programme (“An Apple a Day” Metacognitive Training, Apple-MCT) was added in the intervention group of participants affected by mental disorders. The primary outcome was weight loss. Secondary outcomes included anthropometric, clinical, and metabolic variables. Psychopathology and health-related quality of life were also evaluated in the psychiatric sample. At 12 months, both intervention groups showed a more marked mean decrease in weight (6.7 kg, SD: 3.57) than the TAU group (0.32 kg, SD: 1.96), and a statistically significant improvement in metabolic variables compared with the control groups. Furthermore, the participants affected by mental disorders included in the intervention group reported improved health-related quality of life. Our findings suggest the need to implement integrated interventions based on a dietary protocol, physical activity, and modification of cognitive style in overweight/obese users with mental disorders.

## Introduction

Individuals with severe mental disorders (SMDs) die, on average, 15–20 years earlier than the general population. This pre-mature mortality is mainly due to metabolic and cardiovascular diseases that occur more frequently, are not prevented, and are inadequately identified in this population (1, 2).

Cardiovascular risk factors in individuals with several SMDs—such as schizophrenia spectrum disorder, bipolar disorder, and major depression—include not only common factors, such as “unhealthy” dietary patterns, smoking habits, low levels of physical activity, obesity, hypertension, diabetes, and dyslipidaemia, but also drug-related factors, therapeutic inertia, and poor adherence to prescribed medication (3–7).

The assumption of consuming psychotropic drugs such as antipsychotics, antidepressants, and mood stabilizers seems to be associated with metabolic and clinical disorders, including weight gain, diabetes, dyslipidaemia, and hypertension (4, 8–10). There is a well-documented relationship between clinical/metabolic complications and second-generation antipsychotics, including olanzapine and clozapine, since they are used in the early stages of mental illness (11–19).

The problem of weight gain induced by psychotropic drugs is underestimated in terms of its consequences (8). It can compromise long-term treatment adherence (20) and increase relapse risk (21). Because of the associated metabolic complications, weight gain can negatively impact one's overall quality of life (22, 23) as well as social stigmas associated with mental disorders (24), life expectancy (25), self-esteem, and poorer psychosocial adaptation (26).

Patients in the early phases of schizophrenia and bipolar disorder are at extremely high risk for developing cardiovascular comorbidity; moreover, their metabolic profile worsens quickly (27, 28). Individuals with schizoaffective disorder are more likely to suffer from metabolic syndrome comorbidity than individuals with schizophrenia or other non-affective psychoses (29).

Not only do those affected by psychotic disorders display metabolic problems, but persons affected by depression (compared to non-depressed people) have a significantly greater risk for developing obesity, especially adolescent women (30), in light of the comorbidity of depression with metabolic ailments (31). The link between depression and cardiovascular disease is complex. Major depressive disorder and self-reported depressive symptoms are associated with elevated visceral adipose tissue and subcutaneous adipose tissue (32).

A very recent review (33) investigating the relationship among adipose tissue compartments, inflammation, and cardiovascular risk in depressive disorder emphasized the significant association of depressive symptoms with severe body composition changes starting in early adulthood. Stapel et al. (33) suggested that this group of patients could be predisposed to common physical disorders, such as diabetes mellitus type 2 and cardiovascular diseases. Increased activity of the HPA axis, physical inactivity, poor nourishment, poor adherence to treatment recommendations, and low-grade inflammation might directly or indirectly worsen this vicious cycle, resulting in higher morbidity and mortality rates due to cardiometabolic disorders (33). The same anxiety disorders were observed in frequent co-occurrence with various medical illnesses, with percentages of up to 30% in participants with cardiovascular diseases, 47.0% in those with diabetes mellitus, and vice versa. High rates of medical conditions were reported in samples of participants with anxiety disorders, and greater severity of both anxiety disorders and medical diseases are observed when they coexist (34).

Compared to the general population, individuals suffering from severe psychiatric disorders, especially schizophrenia, tend to engage in a low level of physical activity (35–37), are more inclined to smoke, and exhibit a greater preference for a high-calorie diet (38). This unhealthy lifestyle and non-adherence to treatment over time could be ascribed to a low level of self-regulatory behaviors (39), cognitive flexibility (40, 41), and low levels of self-esteem (42). In recent years, both national and international groups have developed cost-effective screening and monitoring guidelines (17, 43–46), although they are not being implemented in the clinical care of users (47, 48). Based on a review of the evidence that users with serious mental illness (SMI) are at increased risk of CVD and diabetes, the European Psychiatric Association (EPA), supported by the European Association for the Study of Diabetes (EASD) and the European Society of Cardiology (ESC), published a statement regarding the guidelines of ESC and EASD Fourth Joint Task Force of the European Society of Cardiology and Other Societies on Cardiovascular Disease Prevention in Clinical Practice (49). The initiative was aimed at improving the care of users suffering from SMI, initiating cooperation and shared care between different health care professionals to raise the awareness of psychiatrists and primary care physicians who care for patients with SMI for screening and treatment of cardiovascular risk factors and diabetes (50). More recently, a meta-analysis of physical activity interventions and their impact on health outcomes for people with SMI, including schizophrenia-spectrum disorders, major depressive disorder (MDD) and bipolar disorder (51), showed that PA can improve cardiorespiratory fitness, quality of life and depressive symptoms, with effects on depressive symptoms comparable to those of antidepressants and psychotherapy. For schizophrenia-spectrum disorders, much evidence indicates that aerobic physical activity can reduce psychiatric symptoms and improve cognition in various subdomains and cardiorespiratory fitness. In contrast, evidence for the impact on anthropometric measures was inconsistent. Lastly, there was a lack of studies investigating physical activity in bipolar disorder, precluding any definitive recommendations.

Among effective diet programs in clinical populations not affected by psychiatric disorders, some studies used a redistribution of dietary macroelements, from cholesterol and saturated fats to carbohydrates, at a low glycaemic index, based on results obtained from the Optimal Macronutrient Intake Trial, to prevent heart disease (OMNIHeart) (52). Moreover, diet and physical activity modification protocols are widely applied in populations affected by hypertension (53, 54).

At present, most studies on weight management during psychopharmacological treatment include behavioral advice, diet programmes, physical exercise (55), and tailored educational programmes (56). Many studies have used pharmacological or cognitive-behavioral approaches (57) rooted in programmes to change lifestyles to reduce weight gain in individuals with mental illness (58–63).

Our primary aim was to evaluate the effectiveness of a dietary protocol and regular aerobic activity on weight, laboratory, and clinical parameters in participants with and without mental disorders compared to an intervention based on correct lifestyle advice. Additionally, we aimed to evaluate the “add-on” results of a brief metacognitive group programme to enhance the intervention's effectiveness in the sample of overweight/obese users with mental disorders undergoing psychopharmacological treatment.

We hypothesized that (1) the dietary protocol and monitoring of regular aerobic activity would have beneficial effects in participants with and without mental disorders on weight, laboratory, and clinical parameters and would produce outcomes that are superior to advice to improve one's self-regulation of food intake and to engage in more physical activity; (2) integrating a brief, structured group metacognitive intervention could further improve the adhesion of participants affected by mental disorders to maintain metabolic and clinical improvements over time, thereby contributing to better mental health.

## Materials and methods

### Design

**Figure 1:**
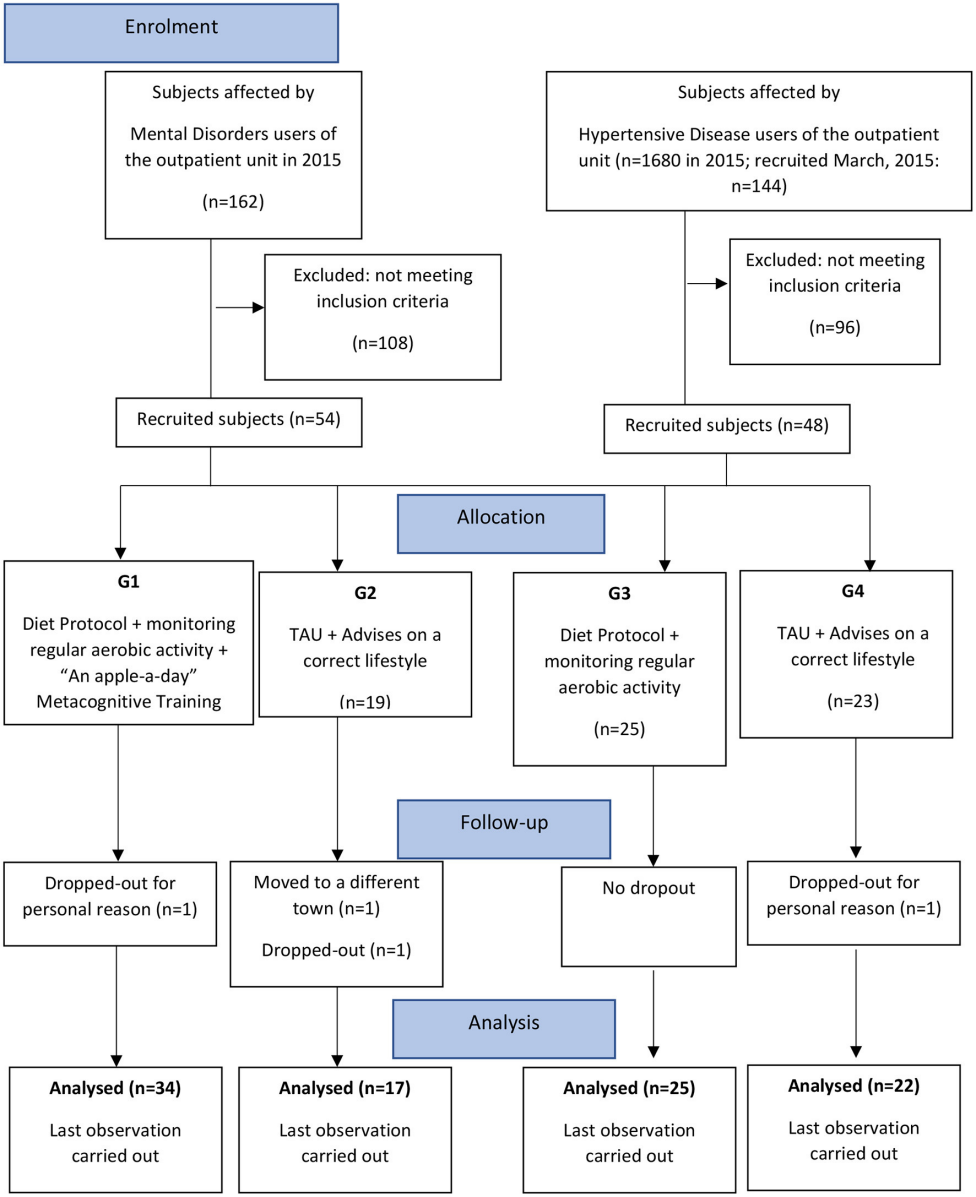
Flow of subjects through the 4-arm study.

The design was a real-world, controlled, pragmatic trial comparing four parallel groups of consecutively allocated participants: those affected by mental disorders undergoing an intervention including a diet protocol, monitoring of regular aerobic activity, and the “An Apple a Day” group Metacognitive Training (Apple-MCT) (G1); participants affected by mental disorders, receiving TAU and advice on a better life-style and bimonthly clinical consultations (G2); participants affected by hypertensive disease undergoing an intervention including a diet protocol and monitoring of regular aerobic activity (G3); and participants affected by hypertensive disease receiving TAU and advice on a better life-style and bimonthly clinical consultations (G4) (Figure 1).

For the psychiatric sample, their assignment was adapted to users' preferences and logistic factors (home distance from the unit, work rotations, difficulty in reaching the unit *via* public transit, etc.). We considered the problems they expressed, mainly when they were offered inclusion in the group intervention and were estimated to attend group sessions.

The inclusion in the protocol did not involve additional fees for the participants.

We carried out the study in compliance with the ethical principles of the Declaration of Helsinki; it was approved by the Ethical Committee of the University of L'Aquila (approval date: 14 October 2014).

### Participants and procedures

All participants were recruited over a 12-month period between January and December 2015 from the TRIP service (Psychosocial Rehabilitation Treatment, Early Interventions in Mental Health Unit) and from the Hypertension and Cardiovascular Prevention Outpatient Unit, both at the University of L'Aquila (Italy).

The participants (aged at least 18) were included according to the presence of at least two of the following:

1) body mass index (BMI) (kg/m^2^) >26;
2) waist circumference (men >102 cm, women >88 cm);
3) hypertriglyceridaemia (≥150 mg/dl);
4) high-density lipoprotein cholesterol (HDLc) (men: <40 mg/dl, women: <50 mg/dl);
5) systolic/diastolic blood pressure levels (≥130/85 mmHg) or diagnosed hypertension;
6) fasting hyperglycaemia (≥100 mg/dl).

The presence of 3 or more of the abovementioned latter elements characterizes metabolic syndrome (MS) (64). MS represents a clustering of factors (hypertension, dyslipidaemia, abdominal obesity, impaired glucose tolerance) predicting an increased risk of cardiovascular disease and stroke (65).

The exclusion criteria for both groups were as follows:

1) severe neurological disorder or intellectual disability or developmental abnormalities or previous head injury;
2) diabetes mellitus, cancer or chronic ailments, prior cardiovascular disease, serum total cholesterol (TC) concentrations >310 mg/dl, triglyceride (TRG) concentrations >350 mg/dl, renal and/or liver insufficiency and any concomitant disease.

All participants included in the psychiatric sample (G1 and G2) received pharmacological treatment: selective serotonin reuptake inhibitor (SSRI) and noradrenergic and specific serotonergic antidepressants (NaSSAs); second-generation antipsychotics; anxiolytics; mood stabilizers; and first-generation antipsychotics (Table 1).

**Table 1:** The demographic and clinical characteristics of the 98 users participating in the study were divided into four groups.

	**Participants affected by mental disorders (*****n*** = **51)**		**Participants affected by hypertensive disease (*****n*** = **47)**	
	**G1 (*n* = 34)**	**G2 (*n* = 1 7)**	**G3 (*n* = 25)**	**G4 (*n* = 22)**
**Gender**, ***n*** **(%)**				
Male	11 (32.4)	3 (17.6)	10 (40)	10 (45.5)
Female	23 (67.6)	14 (82.4)	15 (60)	12 (54.5)
Age, mean (SD)	41.3 (13.4)	43.5 (15.8)	49.1 (12.0)	49.3 (13.8)
Education, years, mean (SD)	13.2 (3.4)	13.4 (3.8)	14.7 (3.1)	13.4 (2.6)
**Marital status** ***n*** **(%)**				
Unmarried/single	23 (67.6)	9 (52.9)	9 (36)	8 (36.5)
Married	10 (29.4)	6 (35.2)	14 (56)	12 (54.5)
Divorced	–	1 (5.9)	2 (8)	1 (4.5)
Widower	1 (3)	1 (5.9)	–	1 (4.5)
**Work status**, ***n*** **(%)**				
Employed	24 (70.6)	13 (76.4)	19 (76)	18 (81.8)
Unemployed	7 (20.6)	2 (11.8)	5 (20)	3 (13.7)
Student	3 (8.8)	2 (11.8)	2 (4.3)	1 (4.5)
BMI overweight range (25– <30)%	20 (58.8)	5 (29.4)	12 (48)	9 (40.9)
BMI obesity range (>30)%	14 (41.2)	12 (70.6)	13 (52)	13 (59.1)
**Diagnosis (DSM-5) (%)**				
Anxiety disorders	16 (47.1)	11 (64.7)	
Depressive disorder	10 (29.4)	4 (23.5)	
Psychotic non-affective disorder	6 (17.6)	2 (11.8)	
Bipolar disorder	2 (5.9)	–	
Length of illness, years, mean (SD)	4.9 (5.1)	3.1 (2.5)	
**Medication (%)**				
SSRI-NaSSAs antidepressants	23 (67.6)	14 (82.3)	
Second generation antipsychotics	5 (14.7)	2 (11.8)	
Anxiolytics	3 (8.8)	1 (5.9)	
Mood stabilizers	2 (5.9)	–	
First-generation antipsychotics	1 (3)		
Polidrug therapy (%)	6 (17.6)		

Waist circumference, height, weight, and blood pressure were measured by trained clinical staff during clinic visits, while fasting plasma lipid levels (triglycerides and low density lipoproteins) and fasting blood glucose levels were measured using regular hospital laboratories. Regarding the metabolic measures, serum low-density lipoprotein cholesterol (LDLc) levels were calculated according to the Friedewald formula (LDLc=TC-(HDL + TRG/5). All analyses were validated by the ISO 9001: 2000 EA: 38 CISQ n. 9122. ASL-IQNET n. IT-65188 quality system. Waist circumference was measured to the nearest 0.1 cm using a standard, inelastic tape maintained on a horizontal plane, with the participant standing with his/her weight distributed evenly on both feet. Height was measured to the nearest 0.1 cm using a wall-mounted stadiometer (without shoes). Weight was measured to the nearest 0.1 kg using standard electronic scales (light clothing without shoes). Blood pressure (BP) was monitored through an OMRON healthcare M2 device while the participant was comfortably seated. Two measurements for SBP/DBP were recorded, and an average was computed.

In this study, BP (i.e., systolic and diastolic BP, SBP/DBP levels) was reported only for participants included in G1 and G2 every 3 months. Those in G3 and G4 consumed anti-hypertensive drugs and were stabilized based on this clinical parameter.

All participants were evaluated at baseline and at the end of treatment (12 months) through a complete electrochemical check.

Dietary monitoring was conducted “face-to-face” by the clinical nutritionist (AnnalisaA.) through meetings every 15 days to check adherence to the dietary protocol and physical activity. The participants included in G1 and G3 were asked to record their weekly physical activity on a form (“My physical activity diary”) about their weekly activity, recorded in hours.

Our study design would investigate psychopathological and psychosocial dimensions only in the group of psychiatric subjects. The cardiovascular prevention outpatient unit clinicians considered that the psychopathological assessment would have taken longer, which is not consistent with the time-sparing organizational goals of the operating outpatient unit. Moreover, they wanted to avoid “psychiatrizing” their users.

### Measures for participants included in the psychiatric groups

Participants affected by mental disorders (G1 and G2) were also evaluated through assessments of psychopathology, health-related quality of life, and personal resources.

The severity of psychopathology was assessed using the Brief Psychiatric Rating Scale-24, BPRS (66) in its Italian version (67). Each symptom on the 24-item scale was rated from 1 to 7 (1 = absence of symptoms; 7 = very severe symptoms). The key score was composed of the total item score.

Health-related quality of life was assessed by the SF-36 Health Survey (68). It is a short-form health survey with only 36 questions. The SF-36 contains eight scaled scores, which are the weighted sums of the questions in their section. Each scale is directly transformed into a 0–100 scale, assuming that each question carries equal weight. The lower the score, the more severe the disability. The higher the score, the less severe the disability; i.e., a score of zero is equivalent to a maximum disability, and a score of 100 is equal to no disability. The eight sections are (1) vitality, (2) physical functioning, (3) bodily pain, (4) general health, (5) physical role functioning, (6) emotional role functioning, (7) social role functioning, and (8) mental health. In the present study, we only considered the “general health” domain.

Self-esteem was assessed by the Self-esteem Rating Scale (SERS) (69). The SERS consists of 40 items rated on a 7-point Likert scale, 20 scored positively and 20 scored negatively, with total scores ranging from −120 to +120. The SERS taps into multiple aspects of self-evaluation, such as overall self-worth, social competence, problem-solving ability, intellectual ability, self-competence, and worth compared to others. Positive scores are indicative of higher self-esteem. The instrument shows a high level of internal consistency (α = 0.97) and good content and factorial validity.

### Interventions

#### Diet protocol

The diet protocol consisted of the modified OMNI-heart programme diet, an individualized, moderately hypocaloric diet based on personal and daily caloric needs; it includes the following:

1) a reduction of 500 kcal/day;
2) daily carbohydrate energy intake of 45%, 50% from whole wheat, and 50% from fruits and vegetables, characterized by a low glycaemic index with a predominance of fructose and sucrose compared to glucose;
3) daily protein energy intake of 25%: 60% from a vegetable source (soy, seitan, beans) and 40% from an animal source (white meat, fish, cheese, milk, and eggs);
4) daily fat energy intake of 30%: 10% Kcal saturated (70), 6% Kcal polyunsaturated fatty acids (omega 3–6), 14% Kcal monounsaturated (extra virgin olive oil);
5) vegetable fiber ≥20 g/die;
6) sodium intake <100 mmol/day, corresponding to a daily intake of 2.4 g;
7) potassium intake >150 mmol/day, corresponding to a daily intake of at least 5 servings of raw fruits and vegetables.

In the present study, the clinical nutritionist (A.A.) applied slight modifications to the basic OMNIHeart dietary protocol, with a carbohydrate decrease and a moderate increase in monounsaturated fatty acids (45% carbohydrates, 25% proteins, and 30% fats in the modified OMNIHeart dietary group and 50% carbohydrates, 25% proteins, and 25% fats in the basic OMNIHeart dietary group). The rationale of this OMNIHeart diet modification was justified by the high rate consumption of carbohydrates in the form of pasta, bread, and sweets (honey and jellies) in the population of L'Aquila in the Abruzzo region. At the same time, there was a relatively low consumption of fats in the form of extra virgin olive oil, which is useful for preventing cardiovascular risk factors. In addition, the increase in monounsaturated fatty acids makes food more palatable to ensure high adherence to the diet programme.

#### Physical activity protocol

Current physical activity levels were assessed by asking the participants about their weekly activity levels as measured using the Metabolic Equivalent of Task (MET) (71). The intensity of physical activity recommended was three METs, equal to a moderate degree (walking) for 3 h per week at 700 METs in accordance with the indications of the World Health Organization (WHO). The MET is a physiological measure expressing the energy cost of physical activities. It is defined as the ratio of metabolic rate (and therefore the rate of energy consumption) during a specific physical activity to a reference metabolic rate, set by convention to 3.5 ml O2/kg/min or 1 kcal/kg/hour.

#### APPLE-MCT

Apple-MCT was a brief, positive, group health-based intervention, followed only by G1, including two modules from the metacognitive training portion (72), (73) using “drill and practice” tasks. The interventions were conducted by a clinical psychologist (L. G.) and a psychiatric rehabilitation technician (A. S.). According to the study protocol, each group was comprised of three to five participants. The Apple-MCT was introduced by a psychoeducational module, including crucial topics for mental and physical health such as sleep–wake cycle regulation, regular physical activity, the timing of meals and meal preparation, good management of comfort eating, and the identification of strengths, new hobbies, and interests, reflecting on what brings happiness. The Apple-MCT included four bimonthly sessions lasting 45–60 min and focused on two specific modules/kinds of content, each alternatively presented in two versions, including different exercises and tasks.

1) Module 3 “Changing beliefs” with the target domain “bias against disconfirmatory evidence” aimed at reducing cognitive inflexibility and the tendency toward overconfidence. In Module 3 (versions A and B), it is explained to the user that it is important to withstand the normal tendency to stick to first impressions, as this response bias can lead to faulty decisions. It is therefore desirable to maintain an open mind. Some negative and dysfunctional beliefs represent severe obstacles to starting and adhering to a diet programme (i.e., “*I am a fickle person and I easily lose motivation*,” “*I do not have the time to stick to a diet and exercise,” “I'm destined to stay fat”*).
2) Module 8 Self-esteem and mood with the target domains “negative cognitive schemata” and “low self-esteem” (versions A and B) aimed at modifying dysfunctional thinking styles, which may contribute to the formation and maintenance of depression and low self-esteem; these are especially correlated with weight control and physical appearance, and lead to difficulty in changing one's eating habits, with an excessive focus on body image or body shape (i.e., “*I am fat and will never be successful in life*,” “*No one will ever love me because of my body and my problems*,” and “*It is all my fault because I neglected my health condition*”).

#### The psychiatric and hypertension treatment as usual group

In the TAU groups, G2 and G4, the participants continued to receive the usual treatment, including regular outpatient assessments, pharmacological treatment, and managing the side effects of medication. Additionally, they were given non-structured information about weight gain and encouraged to limit their food intake and increase the degree to which they exercised.

### Statistical analysis

Descriptive analyses were used to characterize our sample concerning sociodemographic and clinical details. Continuous variables are reported as means (standard deviations), and categorical variables are reported as frequencies (percentages). Baseline comparisons [chi-square, *t*-tests, and one-way analysis of variance (ANOVA)] were performed to assess differences between the psychiatric and medical samples and the four groups. Bonferroni *post-hoc* correction was calculated.

We developed general linear models for repeated measures analyses with a between-subjects factor (G1, G2, G3, G4) and a within-subjects factor (pre-treatment–T0 vs. post-treatment–T1) for physical and metabolic variables. For the variables not fitting the normal distribution, to test the intergroup differences for anthropometric and metabolic variables in the study arms, we used the Kruskal–Wallis test and then made paired comparisons with the *post-hoc* Bonferroni's correction test.

In the psychiatric sample, we employed a general linear model for repeated measures with a between-subjects factor (G1, G2) and a within-subjects factor (pre-treatment–T0 vs. post-treatment–T1) for psychopathological and health-related quality of life variables. Statistical analyses were performed using SPSS 27.0 (SPSS Inc., Chicago, IL, USA). All tests were two-tailed, and *P* < 0.05 was considered significant.

## Results

We recruited a total of 102 people: 54 stabilized participants affected by anxiety disorders, mood, and psychotic disorders according to DSM-5 criteria (74), and 48 participants affected by hypertensive disease.

All participants signed informed written consent forms.

Table 1 describes the final analyzed sample's main demographic and clinical characteristics of 98 subjects.

In the entire sample, the mean age was 45.2 (SD: 13.9) (range: 18–75). The majority of the participants were women (65.3%). There were no statistically significant differences between the two groups (psychiatric and medical participants) concerning sociodemographic variables such as sex, education level, and employment status (Table 1). The medical participants in G3 and G4 were older than those in the psychiatric groups, G1 and G2 [49.23 (SD 12.8) vs. 42.10 (SD 14.10); *t*-test −2.613; *p* = 0.010], the latter showing a higher statistically significant proportion of singletons (62.7 vs. 36.2%; chi-square: 8–156; *p* = 0.043).

No statistically significant differences were found in the proportion of overweight/obese participants included in the four groups (chi-square: 4.357; d.f. 3; *p* = 0.225).

The majority of the participants included in the psychiatric sample were affected by anxiety and depressive disorders (80.4%). According to diagnosis and psychopathological severity, all participants affected by mental disorders were taking psychopharmacological treatments with differences in type and dosage. Regarding G1 and G2, there were no statistically significant differences for the diagnoses and psychopharmacological treatments (Table 1). The participants affected by hypertensive disease were administered hypertensive pharmacological therapies.

### Anthropometric and metabolic variables

At baseline (T0), no statistically significant differences were found among the four groups concerning weight, BMI, and waist circumference.

After 12 months (T1), significant differences over time—but not among the four groups—were found in all measured physical and metabolic variables (Table 2). The significant effects of the interaction time × group (*p* < 0.001) for all the considered variables indicate the intervention's benefit over time, without highlighting differences in the four arms of the study.

**Table 2:** Anthropometric and metabolic variables upon entry into the study (T0) and at the 12-month follow-up (T1).

**Characteristics**	**Participants affected by mental disorders (*****n*** = **51)**				**Participants affected by hypertensive disease (*****n*** = **47)**				***F*** **(group × time interaction)**	***N_2_p*** **(estimated effect size)**
	**G1 (*****n*** = **34)**		**G2 (*****n*** = **17)**		**G3 (*****n*** = **25)**		**G4 (*****n*** = **22)**			
	**T0**	**T1**	**T0**	**T1**	**T0**	**T1**	**T0**	**T1**		
**Anthropometric variables, mean (SD)**										
Weight, kg	81.3 (15.89)	74.2 (14.7)	85.7 (12.7)	85.0 (13.2)	85.30 (14.9)	79 (14.8)	84.9 (12.4)	84.9 (12.0)	Time: 122.281^**^ Group: 1.651 Interaction: 35.016^**^	0.528
BMI, kg/m^2^	30.7 (5.3)	28.0 (5)	32.4 (3.6)	31.8 (3.8)	31 (3.6)	28.7 (3.4)	30.9 (2.4)	30.9 (2.4)	Time: 81.606^**^ Group: 2.045 Interaction: 20.923^**^	0.400
Waist circumference, cm	100.3 (10.7)	94.5 (9.3)	102 (7.3)	101.0 (8)	103 (6.1)	96.5 (6.3)	103.3 (8.6)	102.6 (9.6)	Time: 137.224^**^ Group: 2.107 Interaction: 27.412^**^	0.467
**Lipids, mean (SD)**										
Total cholesterol, mg/dl	229.09 (35.0)	207.7 (27.2)	226.4 (29.3)	223.2 (33.6)	228.44 (23.4)	212 (21.2)	224.95 (23.6)	224.23 (24)	Time: 67.615^**^ Group: 0.348 Interaction: 17.042^**^	0.352
LDLc, mg/dl	140.4 (26.4)	120.97 (17.9)	137.5 (32.6)	137.2 (32.4)	144.96 (22.2)	130.5 (17.4)	147.05 (25.5)	146.6 (25.08)	Time: 38.304^**^ Group: 2.078 Interaction:13.129^**^	0.295
HDLc, mg/dl	43.12 (12.1)	47.03 (11.2)	44.8 (11.0)	44.7 (10.6)	44.7 (9.5)	47.8 (10.3)	40.1 (7.9)	39.3 (8.05)	Time: 32.666^**^ Group: 1.827 Interaction: 20.083^**^	0.391
TRG, mg/dl	176.7 (71.5)	135.8 (46.5)	160.0 (68.1)	154.2 (68.1)	177.8 (65.4)	142.9 (35.0)	169.9 (35.3)	168.8 (34.8)	Time: 28.143^**^ Group: 0.313 Interaction: 7.117^**^	0.185
**Fasting glucose, mean (SD)**										
GLU, mg/dl	97.6 (10)	87.09 (6.9)	93.3 (9)	92.8 (8.6)	97.2 (8.08)	89.5 (5.9)	96.3 (8.7)	96.0 (8.5)	Time: 69.701^**^ Group: 1.114 Interaction: 23.637^**^	0.419

** p = 0.01.

Changes in anthropometric and metabolic variables at the 12-month follow-up (T1) compared to the time of entry into the study (T0) were analyzed.

At 12 months, both intervention groups showed a more marked mean decrease in weight at −6.7 kg (SD: 3.57) than the TAU groups at −0.32 kg (SD: 1.96) (Table 3).

**Table 3:** Mean differences (SD) in anthropometric and metabolic variable changes at the 12-month follow-up (T1) compared to entry into the study (T0).

**Variables**	**Change G1 (T1–T0)**	**Change G2 (T1–T0)**	**Change G3 (T0–T1)**	**Change G4 (T0–T1)**
**Physical, mean (SD)**				
Weight, kg	−7.06 (4.21)	−0.76 (2.27)	−6.24 (2.47)	0.022 (1.67)
BMI, kg/m^2^	−2.65 (1.97)	−0.53 (1.43)	−2.27 (1.04)	0.059 (0.57)
Waist circumference, cm	−5.88 (3.19)	−1.00 (3.18)	−6.48 (2.46)	−0.63 (2.46)
**Lipids, mean (SD)**				
Total cholesterol, mg/dl	−21.32 (16.70)	−3.17 (12.51)	−16.44 (8.82)	−0.72 (3.89)
LDLc, mg/dl	−19.44 (21.06)	−0.35 (2.66)	−14.40 (8.85)	−0.40 (3.63)
HDLc, mg/dl	+3.91 (3.75)	−0.05 (2.46)	+3.12 (1.48)	−0.81 (0.79)
TRG, mg/dl	−40.82 (50.39)	−5.76 (14.45)	−34.88 (42.51)	−1.09 (5.15)
**Fasting glucose, mean (SD)**				
GLU, mg/dl	−10.58 (7.28)	−0.47 (2.62)	−7.64 (5.62)	−0.27 (2.86)

**Figure 2:**
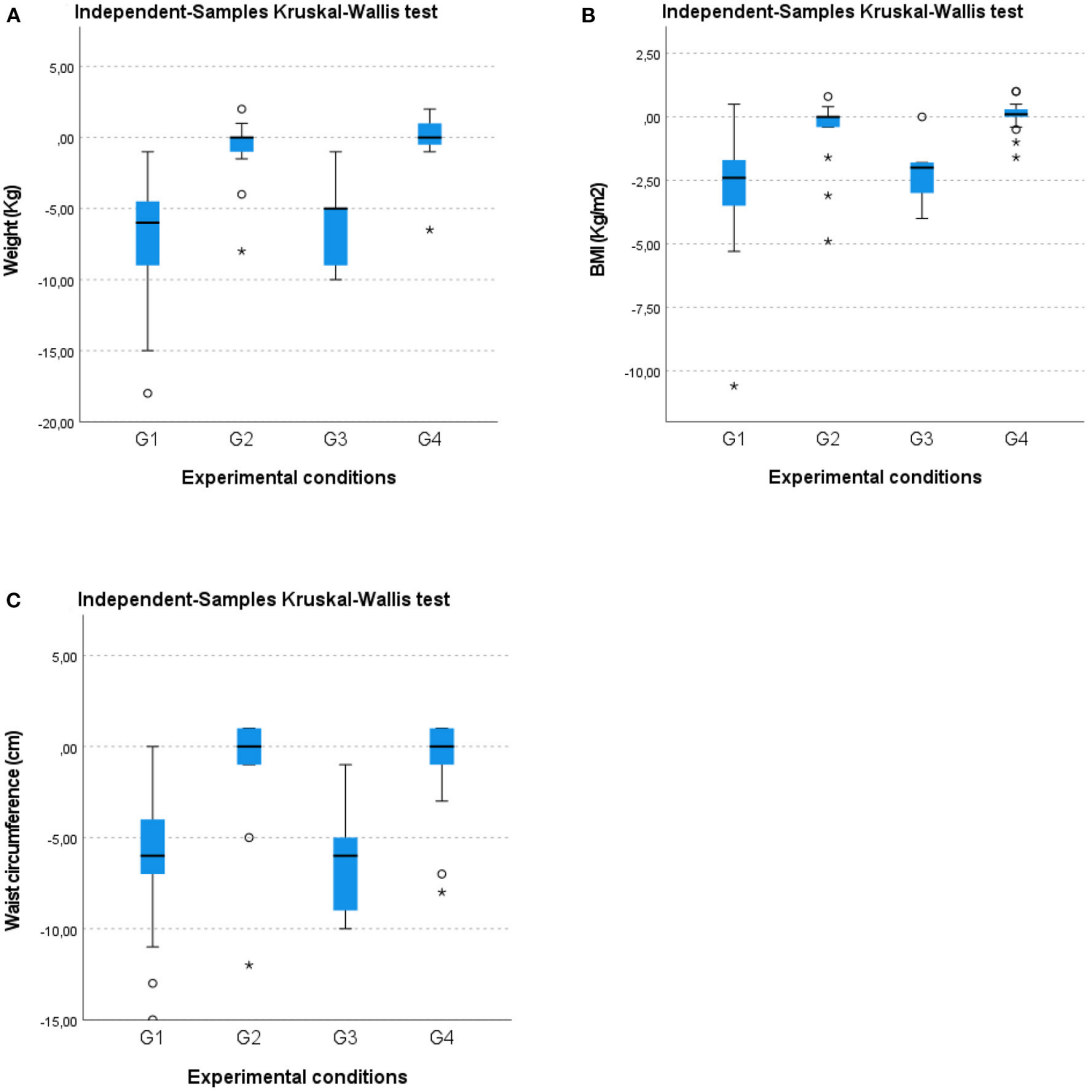
Box-whisker plots showing **(A)** weight (kg), **(B)** BMI (kg/m^2^), and **(C)** waist circumference (cm) changes in the four groups at the 12-month follow-up (T1). O, Outliers; *, Extremes.

A Kruskal–Wallis test and post hoc analysis provided strong evidence of differences between the mean ranks of the two groups (G1 and G3) compared to G2 and G4 at T1 concerning weight [H(3) = 59.811; *p* = 0.00], BMI [H(3) = 50.868; *p* = 0.00], and waist circumference [H(3) = 49.235; *p* = 0.00] reduction (Figures 2A–C). No differences in weight reduction, BMI, or waist circumference were noted between G1 and G3 or between G2 and G4. These results suggest that G1 and G3 exhibited a larger statistically significant improvement than G2 and G4 regarding anthropometric parameters, body weight, waist circumference, and BMI.

**Figure 3:**
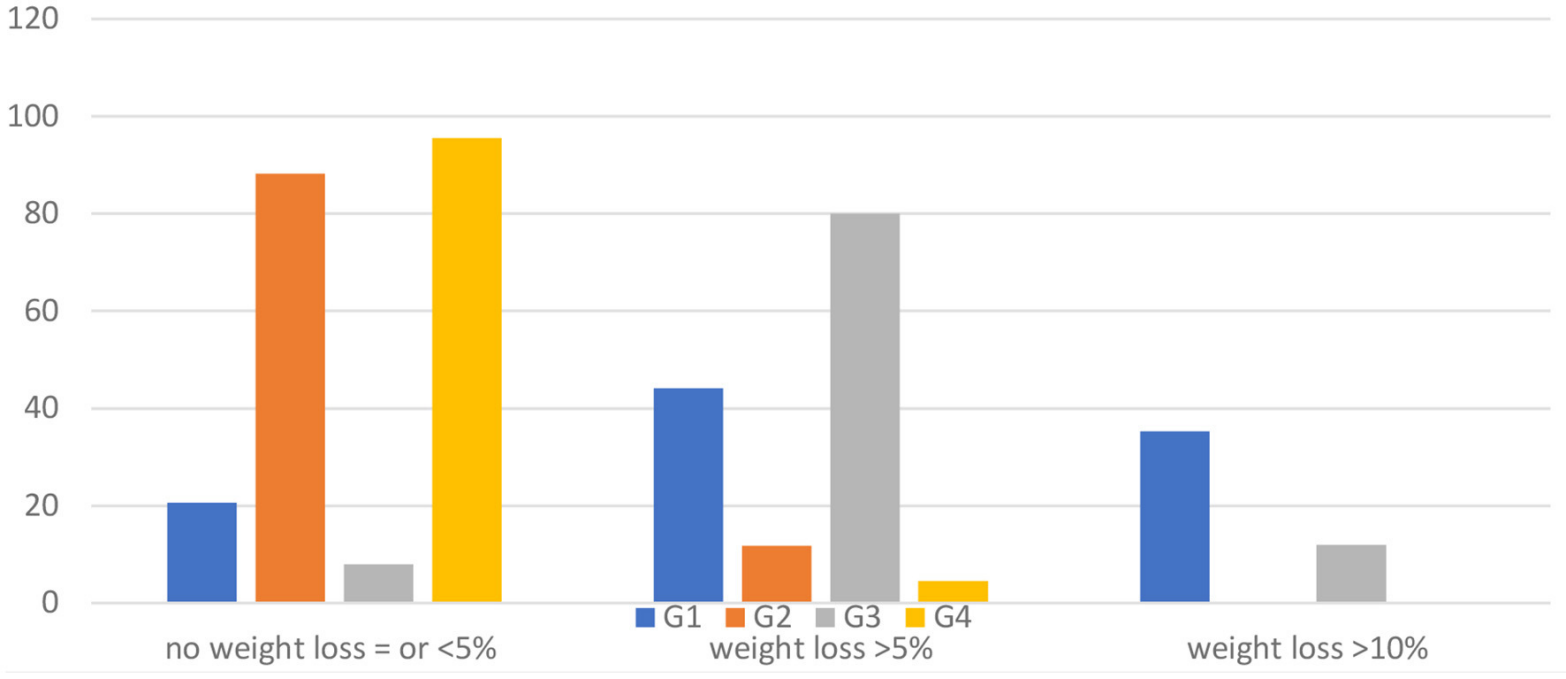
Percentage of weight change at the 12-month follow-up (T1) in the four groups compared to entry in the study (T0).

Figure 3 displays the percentages of participants meeting certain weight-loss thresholds at 12 months in the four groups, showing a significantly different proportion of subjects losing more weight (chi-square: 67.041; d.f. 6; *p* = 0.000). Briefly, the intervention groups G1 and G3 revealed a statistically significant difference in the proportion of participants who lost 5% (59.3%) or 10% (25.4%) of their baseline weight compared to participants included in G2 and G4 who lost 5% (7.7%) or 10% of their baseline weight (0%). Both control groups indicated that 92.3% of the participants recorded a <5% weight loss compared to the intervention groups (15.3%) (chi-square = 56.415; d.f. 2; *p* = 0.000).

**Figure 4:**
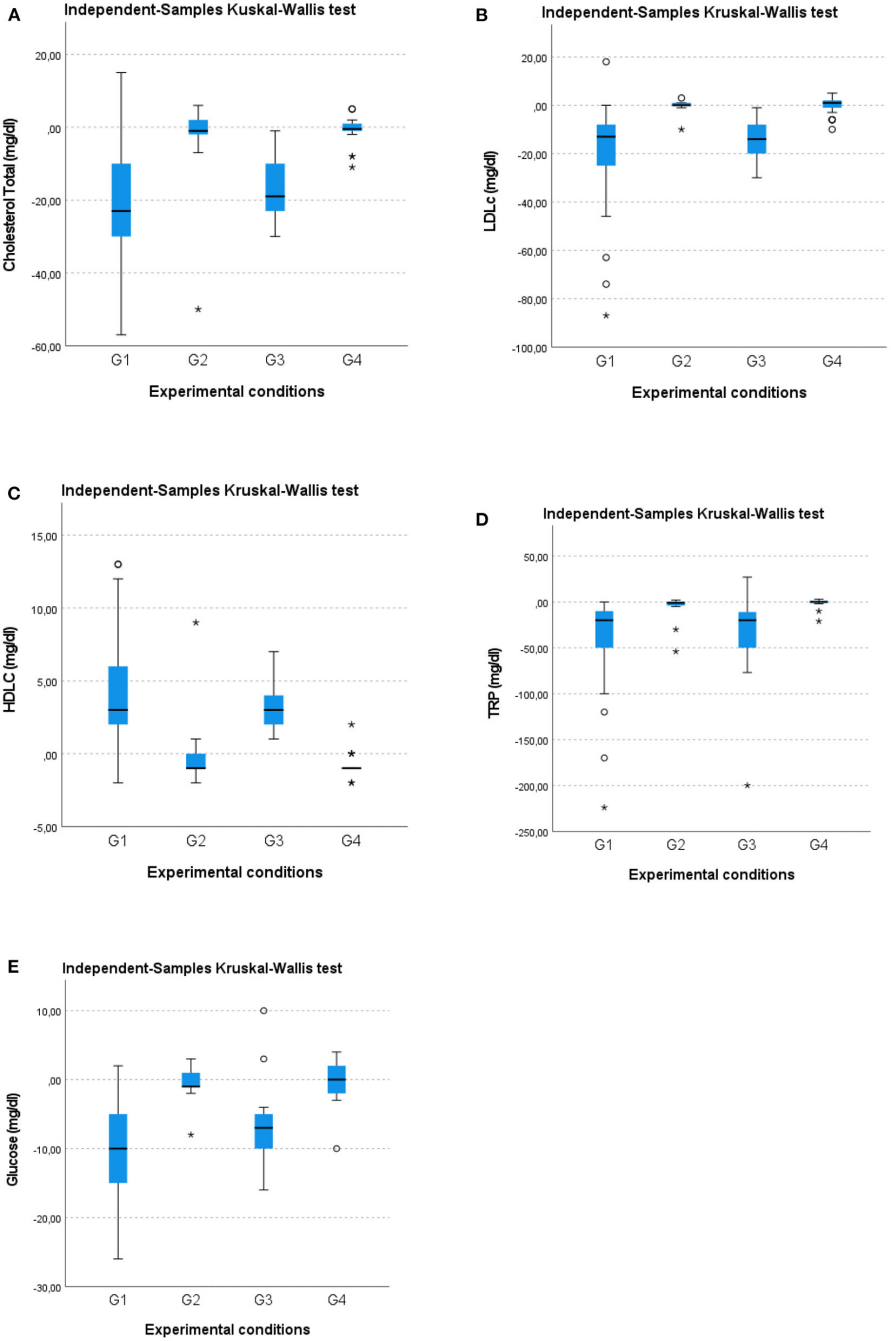
Box-whisker plots showing **(A)** total cholesterol (mg/dl), **(B)** low-density lipoprotein cholesterol, LDLc (mg/dl), **(C)** high-density lipoprotein cholesterol, HDLc (mg/dl), **(D)** triglycerides, TRG (mg/dl), and **(E)** glucose (mg/dl) changes in the four groups at the 12-month follow-up (T1). O, Outliers; *, Extremes.

A Kruskal–Wallis test and *post-hoc* analysis provided strong evidence of differences between the mean ranks of two groups (G1 and G3) compared to G2 and G4 at T1 concerning reduction of total cholesterol (mg/dl) [H(3) = 46.584; *p* = 0.00], LDLc (mg/dl) [H(3) = 55.415; *p* = 0.00], TRG (mg/dl) [H(3) = 46.954; *p* = 0.00], glucose (mg/dl) [H(3) = 50.198; *p* = 0.00] and an increase in HDLc (mg/dl) [H(3) = 54.172; *p* = 0.00; Figures 4A–E. No difference in such metabolic variables was observed between G1 and G3 or between G2 and G4. These results imply that G1 and G3 experienced a larger statistically significant improvement than G2 and G4 in terms of the metabolic variables.

### Clinical measures

#### Blood pressure in the psychiatric sample

At baseline, T0, no significant differences were found among the psychiatric groups concerning SBP [G1 133.24 (SD 7.6) vs. G2 131.1 (9.1); *t*-test for paired samples: *t* = 0.848; *p* = 0.400] and DBP [G1 88.4 (SD 3.9) vs. G2 89.1 (4.4); *t*-test for paired samples: *t* = −0.551; *p* = 0.584].

**Figure 5:**
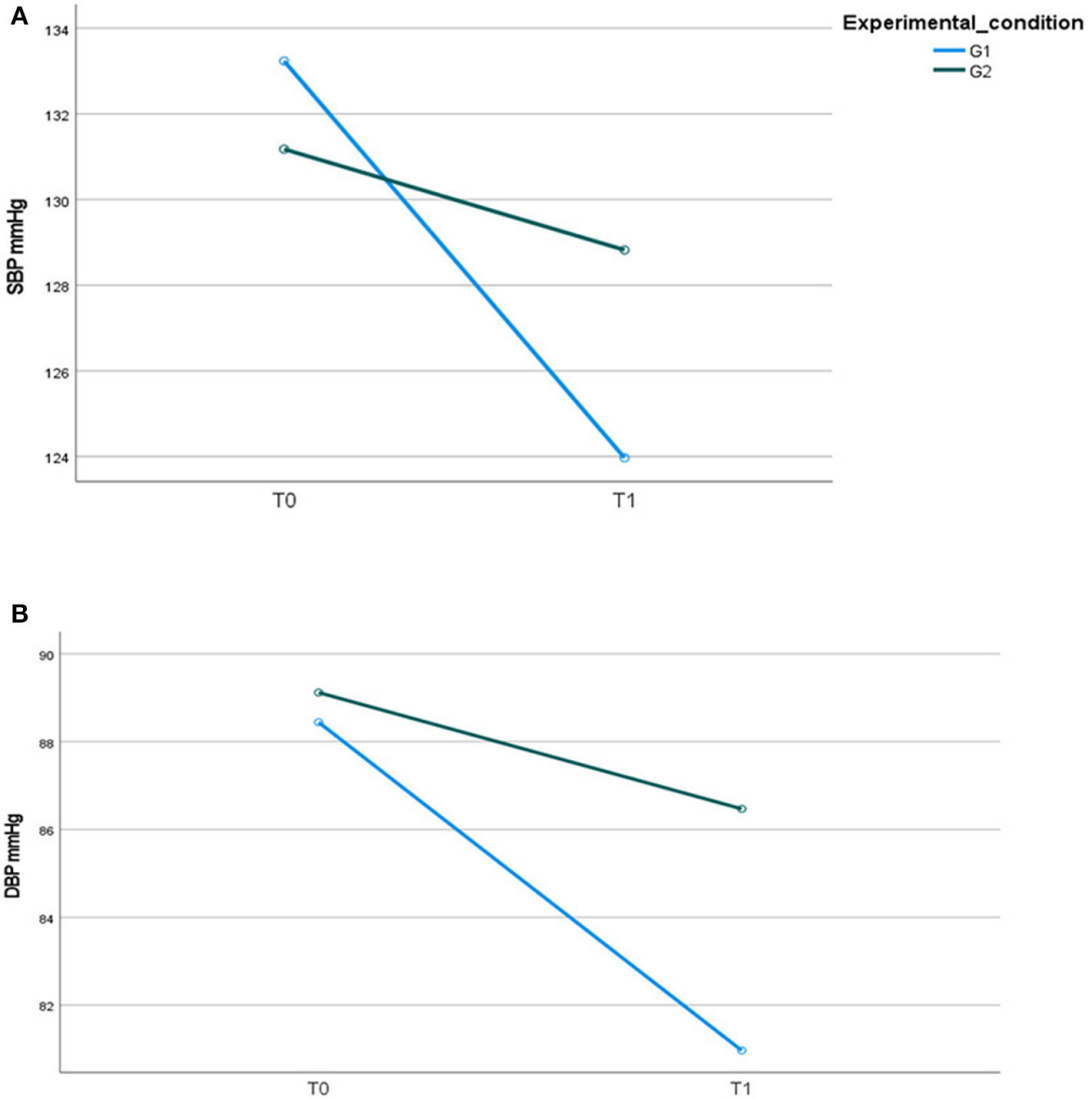
**(A)** Systolic blood pressure, SBP mmHg; **(B)** diastolic blood pressure, DBP mmHg, in the two psychiatric samples at T0 and T1.

After 12 months (T1), significant differences over time—but not between groups—were found for SBP (Figure 5A). At the end of the intervention, for DBP, a change over time with a significant *group for time interaction* (*F* = 13.999; *p* = 0.001; η^2^ = 0.221) was found between the two groups (*F* = 8.611; *p* = 0.005; η^2^ = 0.149), indicating a greater reduction in G1 compared to G2 (Figure 5B).

### Life-Style

The main life-style behavior information (physical activity and smoking) upon entry is outlined in Table 4.

**Table 4:** Physical activity and smoking habits of the participants included in the sample at the time of entry into the study.

	**Participants affected by mental disorders (*****n*** = **51)**		**Participants affected by hypertensive disease (*****n*** = **47)**	
**Variables**	**G1 (*n* = 34)**	**G2 (*n* = 17)**	**G3 (*n* = 25)**	**G4 (*n* = 22)**
**Physical activity (hours/week) T0 (%)**				
No physical activity	22 (64.7)	16 (94)	23 (92)	18 (81.8)
1 h	1 (2.9)	1 (6)	–	3 (13.6)
2 h	11 (32.4)	–	2 (8)	1 (4.6)
**Smoking habits T0 (%)**				
No smoking habits	18 (53)	8 (47)	11 (44)	10 (45.5)
1/2 cigarettes daily	2 (6)	2 (11.8)	–	3 (13.6)
5 cigarettes daily	6 (17.6)	1 (6)	3 (12)	2 (9.1)
10 cigarettes daily	3 (8.8)	3 (17.6)	8 (32)	3 (13.6)
20 cigarettes daily	5 (14.6)	3 (17.6)	3 (12)	4 (18.2)

Regarding physical exercise, all participants practiced low physical activity (average of 2 h weekly < 3 MET). The majority of the participants (80.6%) did not engage in any physical activity, except the participants included in G1, who showed significantly higher activity (chi-square: 18.955; df 6; *p* = 0.004). The four groups did not exhibit statistically significant differences in the proportion of smokers compared to non-smokers (chi-square: 0.556; df 3; *p* = 0.906).

Regarding eating habits, no statistically significant differences were found among the four groups at the time of entry into the study. All participants reported irregular eating habits (low consumption of fruits, vegetables, and olive oil; high consumption of sugar, alcohol, and saturated fats).

**Figure 6:**
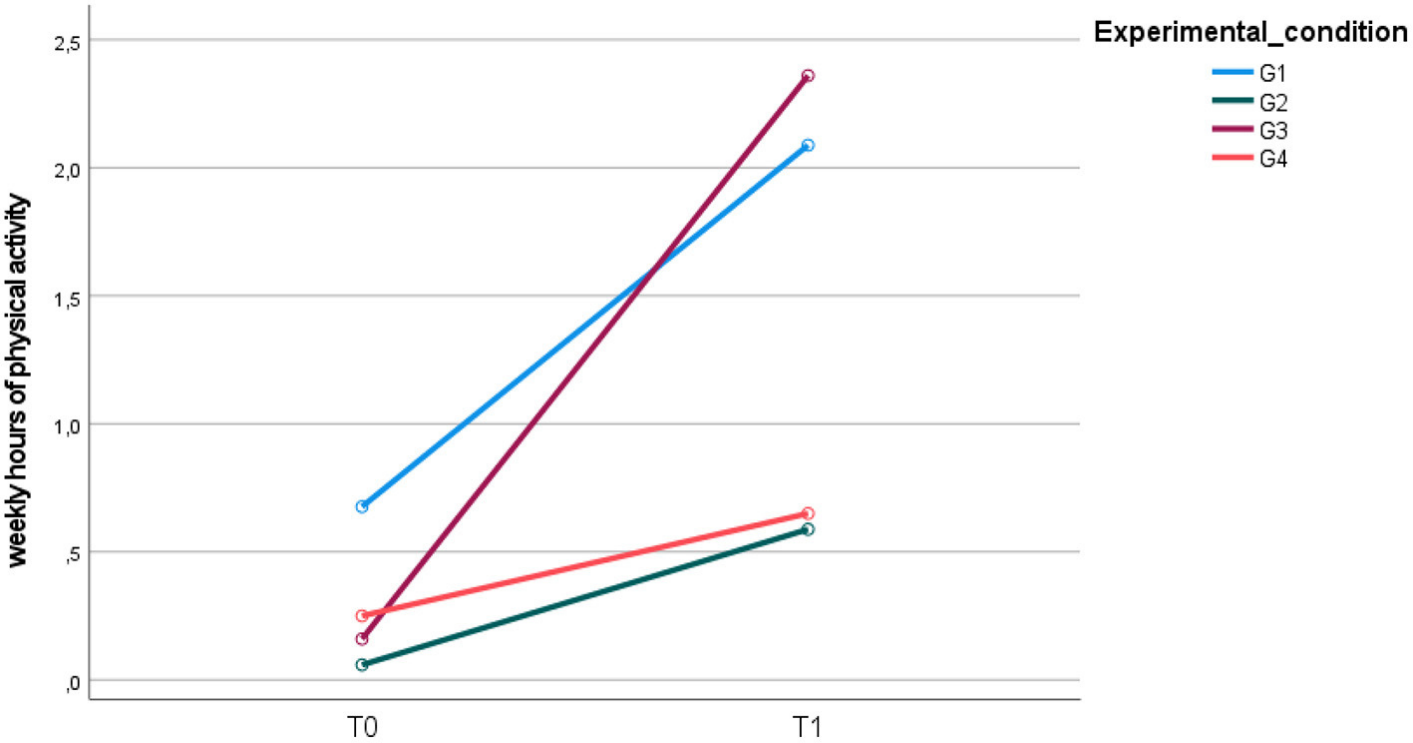
Mean weekly hours of physical activity upon entry into the study (T0) and at the 12-month follow-up (T1).

At the end of the intervention, concerning physical activity, a significant change over time (group for time interaction *F* = 26.901; *p* = 0.000; η^2^ =0.467) was observed in the four groups (Figure 6).

At the end of the study, significant differences were found between G1 and G2 (95% CI: 0.52, 1.50; *p* = 0.000), G1 and G4 (95% CI: 0.43, 1.44; *p* = 0.000), G2 and G3 (95% CI: −1.5, −0.37; *p* = 0.000), and G3 and G4 (95% CI: 0.27, 1.35; *p* = 0.001), showing a statistically significant increase in physical activity for both G1 and G3 compared to G2 and G4 (Figure 6).

No statistically significant differences were observed in smoking habits at T1 compared to T0.

Concerning eating habits, diet improvements can be mainly inferred from weight changes at T1.

### Psychopathology

At baseline, no statistically significant differences were found between the G1 and G2 groups for BPRS total scores. There was a psychopathological improvement at the end of treatment with a significant *group for time interaction* and a decrease in the BPRS total score for both groups (Table 5).

**Table 5:** Psychopathological, health-related quality of life, and personal resources in G1 and G2 at T0 and T1.

	**Participants affected by mental disorders**					
	**G1 (*****n*** = **34)**		**G2 (*****n*** = **17)**		***F*** **-value**	**η^2^p**
	**T0**	**T1**	**T0**	**T1**		
**Psychopathology, mean (SD)**						
Brief psychiatric rating scale-24, BPRS, total score	59.9 (5.8)	51.5 (4.7)	57.5 (4.8)	51.8 (5.5)	Time 138.568^**^ Group 0.508 Interaction 5.359^*^	0.099
**Health-Related quality of Life, mean (SD)**						
Health-related quality of life, SF-36 self-perception general health	46.2 (10.2)	59.8 (8.6)	44.7 (9.9)	48.2 (11.5)	Time 134.427^**^ Group 5.237^*^ Interaction 46.419^**^	0.486
**Personal resources variable, mean (SD)**						
Self-Esteem rating scale, SERS	25.0 (30.6)	41.3 (26.4)	17.3 (26.4)	35.2 (21.6)	Time 66.966^**^ Group 0.134 Interaction 0.781	0.003

* p ≤ 0.05.

** p ≤ 0.01.

### Health-related quality of life

**Figure 7:**
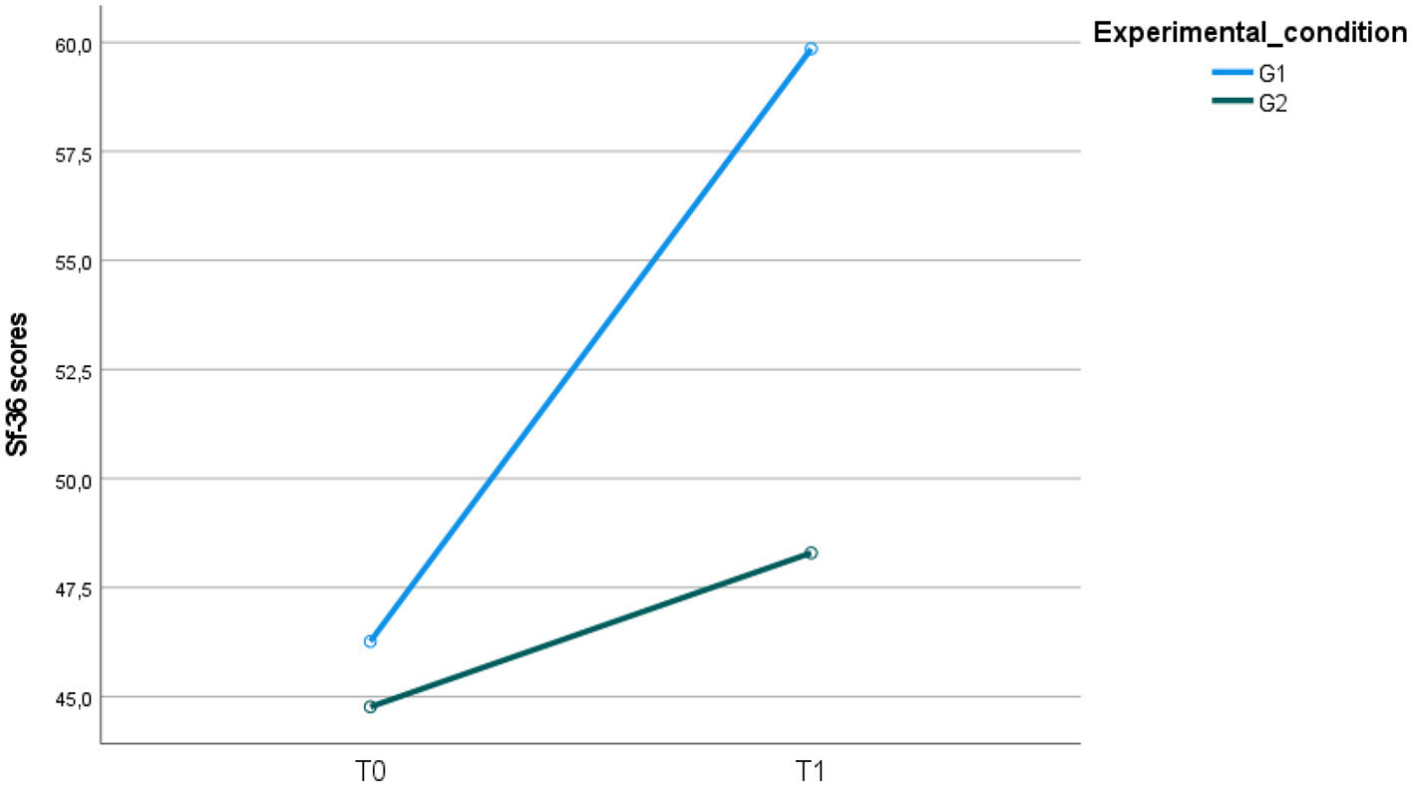
Health-related quality of life (SF-36) scores in the two psychiatric samples (G1, G2) at T0 and T1.

At baseline, no significant differences were found between the G1 and G2 groups concerning health-related quality of life, evaluated through the SF-36. At the end of the intervention, health-related quality of life scores changed for the two groups with a significant *group for time interaction*. Participants in G1 experienced better improvements in their health-related quality of life SF-36 scores than participants included in G2 (Figure 7).

### Self-esteem

At baseline, low values of self-esteem, evaluated by the SERS, were reported by all participants included in G1 and G2, without significant differences between the two groups. At the end of the intervention, participants in both groups revealed increased SERS scores regarding self-esteem levels without a significant change over time and between the two groups (Table 5).

## Discussion

To the best of our knowledge, the present study is the first Italian real-world pragmatic controlled study to assess the effectiveness of a multi-component intervention based on a modified OMNI-heart programme diet and physical activity, including a group metacognitive programme, in a sample of overweight/obese users of a psychiatric outpatient service.

The study showed the same effectiveness for overweight/obese participants affected by hypertension and overweight/obese participants affected by mental illness undergoing psychopharmacological treatment, with an added transdiagnostic brief metacognitive group programme, compared to an intervention limited to recommendations on how to live a better life-style. Moreover, at the 12-month follow-up, participants in this intervention group exhibited increased health-related quality of life compared to participants receiving only recommendations on a healthy life-style.

Our findings align with previous results about reducing body weight, modifying metabolic parameters, and life-style in both populations of psychiatric and hypertensive individuals using a multi-component intervention (53–55). Our overall cardiovascular risk reduction is comparable to prior multi-component studies in the psychiatric (61, 75) and general populations (76). The effects of the macro-element redistribution were investigated, which concerned almost all the other cardiovascular risk factors including TC, LDLc, HDLc, TRG, and fasting GLU; these are probably more reliable and robust, albeit with substantially quantitative differences. In particular, the impressive reduction of serum TRG levels in both intervention groups could be due to an array of independent factors such as the reduction of carbohydrate energy, concomitant increases in protein, and perhaps to a greater extent in unsaturated fats (77, 78). The observed reduction in fasting glycaemia levels may also be due to the combination of low carbohydrates, high protein, and high-unsaturated fats in association with the moderately hypocaloric diet plus physical exercise.

Our results are encouraging and identify a new “target” of life-style interventions, not only for persons affected by severe mental illness, but also for a transdiagnostic group receiving mental health care, as proposed in a recent protocol for young people (79). Reduced weight loss in the range of 2% (80) to 4.2% (81) was reported in adults with severe mental illness, while we observed a mean weight loss of −8.6% for our psychiatric intervention group. Compared to studies including only psychotic populations, our findings seem to show a more marked net weight loss in the intervention group, presumably justified by a larger share of participants affected by anxiety and depressive disorders.

The length, the multi-component nature of our study, and the strict monitoring at 12 months could justify our results as better compared to the findings of a shorter 3 month intervention based only on an educational programme that demonstrated effectiveness only in increasing physical activity, but not for clinical and laboratory parameters (82). The critical aspect of the duration of life-style interventions of 12 months or more for treating overweight and obese people with serious mental illness was already stressed (83, 84), and their systematic reviews and meta-analyses reported that these interventions achieve more consistent outcomes.

Length does not seem to be the only critical variable in the effectiveness of life-style interventions. Our “face-to-face” intervention seems more promising than a multimodal web-based intervention administered by nurses to manage life-style changes in participants affected by severe mental illness (85). Using a web tool in the multi-modal, patient-centered life-style intervention did not seem to improve waist circumference and metabolic health after 12 months in a Dutch sample (85).

In a multi-component intervention, the “active ingredients” are difficult to identify. The added intervention for the psychiatric intervention group, including a group metacognitive programme, could have contributed to the intervention's effectiveness in the psychiatric group. We can hypothesize that the “An Apple a Day” metacognitive group intervention could have contributed to the outcomes, improving cognitive flexibility, a crucial variable specifically influencing self-regulatory behavior associated with healthier eating (86). Self-regulatory skills applied to controlled eating may be a far more critical factor than knowledge of appropriate nutrition principles in the behavioral treatment of obesity (87, 88). Additionally, the increased physical activity per week of the intervention group, favored by frequent checks leading to high user compliance (89), could have contributed to the outcomes. The health benefits of physical activity include the impact of exercise on cognitive functioning in general (90) and psychiatric populations (91).

At the 12-month follow-up, all participants affected by mental disorders improved their psychopathological conditions and self-esteem since they adhered to their pharmacological treatment and were compliant with the monthly consultations. Our intervention in the psychiatric group did not show specific symptomatologic benefits. Regarding psychopathology, our results are partially similar to those of a previous study on individuals with severe mental illness (81). The study revealed significant improvement in total activity, weight, abdominal girth, systolic blood pressure, and HDL cholesterol following the Multidisciplinary Life-style enhancing Treatment for Inpatients (MULTI) compared to treatment as usual (TAU). Despite such improvement, the participants included in MULTI did not display psychopathological progress after 18 months (81). In addition, similar results were reported by Kahl et al. (92) in a randomized pilot study: they showed the favorable additional effect of a 6-week structured, supervised exercise program on visceral, in particular epicardial and subcutaneous, adipose tissue in users with MDD undergoing cognitive behavioral therapy, with significant improvement of factors constituting the metabolic syndrome.

A reduction in symptom severity was reported in physical activity interventions (35, 51, 93), which is not in line with our findings. A systematic review and meta-analysis of the future risk of mental illness indicated that the incidence of mental disorders and suicidality was inversely related to fitness (94).

Our psychiatric intervention sample showed significantly improved health-related quality of life compared to the controls, confirming recent findings (75, 88, 95). Improvements in body image and health-related quality of life seem closely linked to changes in weight (89).

However, our findings did not confirm increased psychological wellbeing in terms of self-esteem in our intervention group as an outcome frequently reported in life-style interventions (75, 96). Surprisingly, the participants in our psychiatric group did not display improved self-esteem, which was found to be inversely correlated with weight gain and good psychosocial adaptation (26).

The weight control issue is overwhelmingly salient in society and of great relevance and concern, also following the COVID-19 pandemic (97, 98). A general population study demonstrated that 22% of American adults gained weight during the COVID-19 pandemic. Lack of sleep, decreased physical activity, snacking after dinner, and eating in response to stress seemed to be behaviors tied to weight gain during self-quarantine (97). During the Italian COVID-19 lockdown, the perception of weight gain was observed in 48.6% of the general Italian population (99). More than 40% reported that they have gained weight to a slight extent, while 8.3% of the studied population said they have gained weight to a high extent. Prevention and management of obesity require consumption of a healthy and energy-balanced diet and adequate physical activity levels (100, 101).

As a pandemic-related physical health change, weight gain was also registered in psychiatric samples, with a greater impact than on the general population (102).

### Strengths and limitations

To the best of our knowledge, no intervention studies have been conducted in psychiatric populations using an integrated intervention based on diet and physical activity programmes and metacognitive modules. The only experience reported was related to cardiac rehabilitation participants included in group metacognitive therapy (six sessions). The intervention successfully improved depression and anxiety compared with usual care, leading to more significant reductions in unhelpful metacognition and repetitive negative thinking (103).

Second, the strength of our study was based on the multi-component and transdiagnostic structure of our intervention, which was well-accepted by our participants. Beyond the diagnosis, from a comprehensive early intervention perspective, the protocol aimed to reduce weight and cardiovascular risk factors such as hyperglycaemia, dyslipidaemia, hypertension, and poor physical activity, all the more reason given the overweight/obese individuals already present and a source of concern for the users. All participants showed good adherence to treatment and reported being very glad to be offered an “extra service” to improve their physical health without any cost.

Our study has several main methodological limitations.

First, our study was a real-world pragmatic trial taking into account psychiatric users' needs and logistic factors. During the informed consent process, the clinicians informed the participants affected by mental disorders that they would have to take part in weekly group sessions. Working or living far away from the site of our service seemed very difficult for some participants. Therefore, they were allocated to the “control” group.

Second, we used an exclusive univariate analytical approach without calculating the power and sample size due to the study's exploratory nature.

Third, the psychiatric sample, including psychopathologically stable participants, had different diagnoses and received different psychopharmacological treatments. Most of them (~80%) were affected by depression and anxiety disorders and treated with SSRIs. The remaining 20%, affected by psychotic disorders, were treated with atypical antipsychotics. Although with varying degrees of severity, the impact of antidepressants and antipsychotics on weight seems sufficiently homogeneous, with an increase in body weight while taking these drugs (8, 104).

The weekly self-report of dietary and physical activity constituted a further limitation for participants in the intervention groups; every 15 days, during the clinical check-up, the clinical nutritionist (A. A.) weighed the participants based on the interventions. However, adherence to the physical activity protocol relied upon the users' statements only.

## Conclusions

The study showed significant benefits of our intervention, including a modified OMNIHeart dietary protocol, in terms of percentage of weight reduction, improvement of metabolic parameters, as recently stressed by Volpe et al. (105), and increased physical activity for both our users and psychiatric and medical subjects. For the psychiatric intervention group, which experienced better health-related quality of life, these differences were found irrespective of medication in an overweight/obese population already presenting with a consistent cardiovascular risk. Life-style interventions can help to manage the physical and mental health symptoms of people affected by psychiatric disorders (106). Alongside medication, a range of psychosocial interventions and behavioral weight management needs to be included to achieve a full and sustained recovery for persons impacted by mental illnesses.

## Data availability statement

The raw data supporting the conclusions of this article will be made available by the authors, without undue reservation.

## Ethics statement

The studies involving human participants were reviewed and approved by Ethical Committee of the University of L'Aquila (approval date: 14 October 2014). The patients/participants provided their written informed consent to participate in this study.

## Author contributions

LG, RR, and MC contributed to the design. VB, SM, and AS contributed to data acquisition. LG, RR, MC, CF, and SN participated in the analysis and interpretation. AAg carried out nutritional consultations for all patients involved in the study. CF and AAl contributed to the collection of clinical and metabolic data of hypertensive patients from the division of internal medicine and nephrology. All authors contributed to the manuscript, revised the work, agree to be accountable for all aspects of the work in ensuring that questions related to the accuracy or integrity of any part of the work are appropriately investigated and resolved, read, and approved the manuscript.

## Conflict of interest

The authors declare that the research was conducted in the absence of any commercial or financial relationships that could be construed as a potential conflict of interest.

## Publisher's note

All claims expressed in this article are solely those of the authors and do not necessarily represent those of their affiliated organizations, or those of the publisher, the editors and the reviewers. Any product that may be evaluated in this article, or claim that may be made by its manufacturer, is not guaranteed or endorsed by the publisher.
